# Predicting global tuna vulnerabilities with spatial, economic, biological and climatic considerations

**DOI:** 10.1038/s41598-018-28805-z

**Published:** 2018-07-12

**Authors:** Alex Tidd, Julia L. Blanchard, Laurence Kell, Reg A. Watson

**Affiliations:** 1 Centre for Marine Socioecology, University of Tasmania, GPO Box 252-49, Hobart, Tasmania 7001 Australia; 2 Institute for Marine and Antarctic Studies, Centre for Fisheries and Aquaculture, University Tasmania, Private Bag 49, Hobart, 7001 Tasmania Australia; 3 Galway-Mayo Institute of Technology (GMIT), Old Dublin Road, Galway, Ireland; 4 Centre for Environmental Policy, Imperial College London, London, SW7 1NE UK

## Abstract

Overfishing impacts the three pillars of sustainability: social, ecological and economic. Tuna represent a significant part of the global seafood market with an annual value exceeding USD$42B and are vulnerable to overfishing. Our understanding of how social and economic drivers contribute to overexploitation is not well developed. We address this problem by integrating social, ecological and economic indicators to help predict changes in exploitation status, namely fishing mortality relative to the level that would support the maximum sustainable yield (F/F_MSY_). To do this we examined F/F_MSY_ for 23 stocks exploited by more than 80 states across the world’s oceans. Low-HDI countries were most at risk of overexploitation of the tuna stocks we examined and increases in economic and social development were not always associated with improved stock status. In the short-term frozen price was a dominant predictor of F/F_MSY_ providing a positive link between the market dynamics and the quantity of fish landed. Given the dependence on seafood in low-income regions, improved measures to safeguard against fisheries overexploitation in the face of global change and uncertainty are needed.

## Introduction

The 2005 World Summit on Social Development identified three main sustainable development goals, related to economic development, social development and environmental protection^1^, these are often referred to as the Three Pillars of Sustainability. Where sustainability is the ability of biological systems to remain diverse and productive indefinitely. Ensuring sustainability across ecological, social and economic dimensions is a cornerstone of international Sustainable Development policy (United Nations Sustainable Development Goals) and Blue Growth initiatives^2,3^.

Fish and seafood are the largest single animal-based food production sector (FISHSTAT, 2017) and provide 4.3 billion people worldwide with 15–20% of their intake of animal protein and in some countries over 50%^4^. The demand for fish continues to increase (4% per year^5^), driven by a growing population (1.18% per year)^6^, technological innovations and the availability of more disposable income. At least 30% of large commercial fish stocks are currently classified as overexploited^2^. Although this is an improvement over previous decades, for many regions of the world — particularly poorer countries with small-scale fisheries — the sustainability status of fisheries and even the amount of fishing happening is uncertain^7^. Further, changes to already heavily exploited systems — mediated by climate, or changes in demand from population growth and wealth — risk fisheries collapses or significant increases in the price of fish products. Given the food security, livelihoods and nutritional importance of fish for so many people, effectively managed fisheries is central to many sustainable development goals^8^.

Tuna represent a significant part of the global fish and seafood economy with an annual value of USD$42.2 billion^9^. Tuna also play an important role in the health and functioning of the ecosystem^10^ and across all sectors of a wider fishing community necessitating improved management to maintain fish stocks in a healthy state^9^. Because of the high value of tuna stocks they are subject to high fishing pressure and there is growing concern about (i) the risks of failing to achieve fisheries and conservation objectives, (ii) the ability to implement recovery plans for depleted stocks and (iii) effective protection for those that are vulnerable to overfishing^11^.

While some progress has been made in developing a precautionary approach to fisheries management for tuna by developing limit reference points to indicate overfishing^12^, this has, however, been mainly confined to biological elements^13^. Although more integrated approaches for addressing the combined ecological, social and economic risks of overfishing have been called for over the past decade^13,14^. This normally requires targets and limits related to economic and social factors in order to effectively manage a fishery.

Tuna Regional Fisheries Management Organisations (tRFMOs) represent international organisations with fishing interests in a specific area (see Figure S1) and most have adopted Maximum Sustainable Yield (MSY) as a management target defined as the largest average catch of a species that can be taken over time that guarantees that the resource is not depleted. MSY is, however, notoriously difficult to estimate and generally relies on equilibrium model assumptions and/or data from stocks that have already exceeded the level (B_MSY_) that supports MSY^15–18^. The management performance of each tuna fishery is monitored by a set of indicators to report on the progress in achieving F_MSY_ (the fishing mortality level consistent with achieving maximum sustainable yield) and B_MSY_ (Spawning stock biomass or Total biomass that results from fishing at F_MSY_ for a sustained period of time)^19^. A major challenge, however, is understanding how the sustainability of fisheries is affected by exernal forces such as climate change^20^ and shocks such as market fluctuations or environmental variability^21^.

Over the last two decades there have been significant changes in fuel costs, fish prices, global warming, technological change (i.e. introduction of gears such as Fish Aggregation Devices, FADs), and changes in adult tuna stock biomass^22^. All of these factors have a cumulative effect on the operating costs of fleets and thus their spatial behaviour^23^. For tuna stocks, past exploitation levels and management measures have shown to be as important as the links between life history, market price and vulnerability to overexploitation^11^. Although a composite index of fisheries management at the country-level has shown to be positively related to factors such as countries’ gross domestic product^24^ an integrated understanding of how these drivers connect to environmental with economic and biological variables for tuna stocks is currently missing.

Here we examine whether tends in tuna stock status, as measured by F/F_MSY_, are related to the economic and social development of countries (Human Development Index, HDI) to identify whether some countries are more risk of overexploitation. We then develop statistical models to explore how stock status could be affected by different types of short-term shocks based on the relationships between F/F_MSY_ with economic fluctuations (e.g. fish prices and fuel price), social (fleet diversity/fishing activity – knowledge transfer) and climatic variability (e.g. North Atlantic Oscillation Index (NAO) and Southern Oscillation index (SOI)). Time series of economic, climatic and spatial indices were available for more than 23 years. As these indicators are potentially correlated, we constructed ridge regression models (see Methods and Materials) and used these to assess the sensitivity of F/F_MSY_ for tuna stock to each driver of change.

## Results and Discussion

### Trends in F/F_MSY_ and HDI

**Figure 1:**
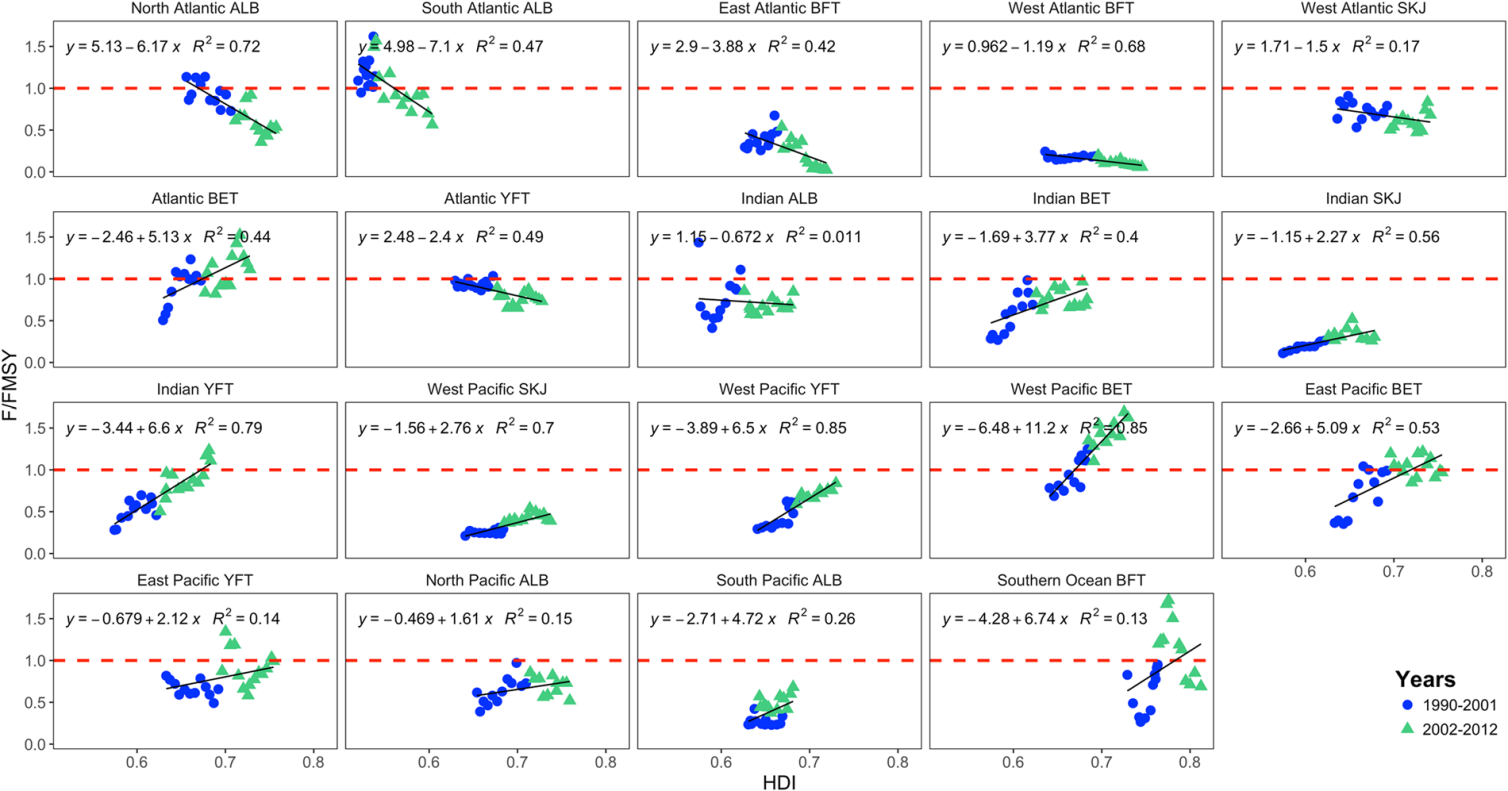
HDI versus F/F_MSY_ for all stocks studied (The blue dots refer to the year period 1990–2001 and the green triangles 2002–2012. The red dashed line indicates fishing mortality that would provide F/F_MSY_.

Our results show that low-HDI countries such as those in the Indian and Pacific oceans are vulnerable to overfishing e.g. western Pacific bigeye tuna and Indian Ocean yellowfin tuna (Fig. 1). Countries within these regions are some of the poorest in the world and rely on tuna for their diet and poverty reduction. Over time these regions have realised the potential of their resource i.e. an increase in human development has coincided with an increase in the ratio of F/F_MSY_ for tuna. In high HDI countries (e.g. in the Atlantic) the opposite observation can be seen, whereby in early years the stocks were subject to overfishing (e.g. North and South Atlantic albacore (Fig. 1)). These finding are consistent with recent work showing that stocks with management in place were less likely to be over exploited^11^. In these countries over time F/F_MSY_ has declined in line with increases in HDI and possibly with socio-economic responsibilities towards overfishing. In high HDI countries, fishers have other livelihood opportunities and/or subsidies^24^. Controlling and managing fisheries in low HDI regions will therefore be crucial especially with rising fuel costs, fluctuating stock levels, changeable market conditions and poor international regulations which are a driver for illegal, unreported and unregulated (IUU) fishing^25^.

### Modelling multiple drivers of variability

Though natural variability is an inherent feature of fisheries that cannot be removed by management it is possible, however, to minimize the effects of such variability through management strategies that deal with the inherent risks and uncertainty^26,27^. Of course, it is impossible to account for all sources of uncertainty, but each process needs to be understood better e.g. how stocks and ecosystems fluctuate along with variable fish prices that affect fishers’ income in the short and long term in response to management and natural variation^28^, as do the sources of uncertainty. The development of cost effective approaches to predict future events can be a valuable tool for conservation and preventative management action^29^.

**Figure 2:**
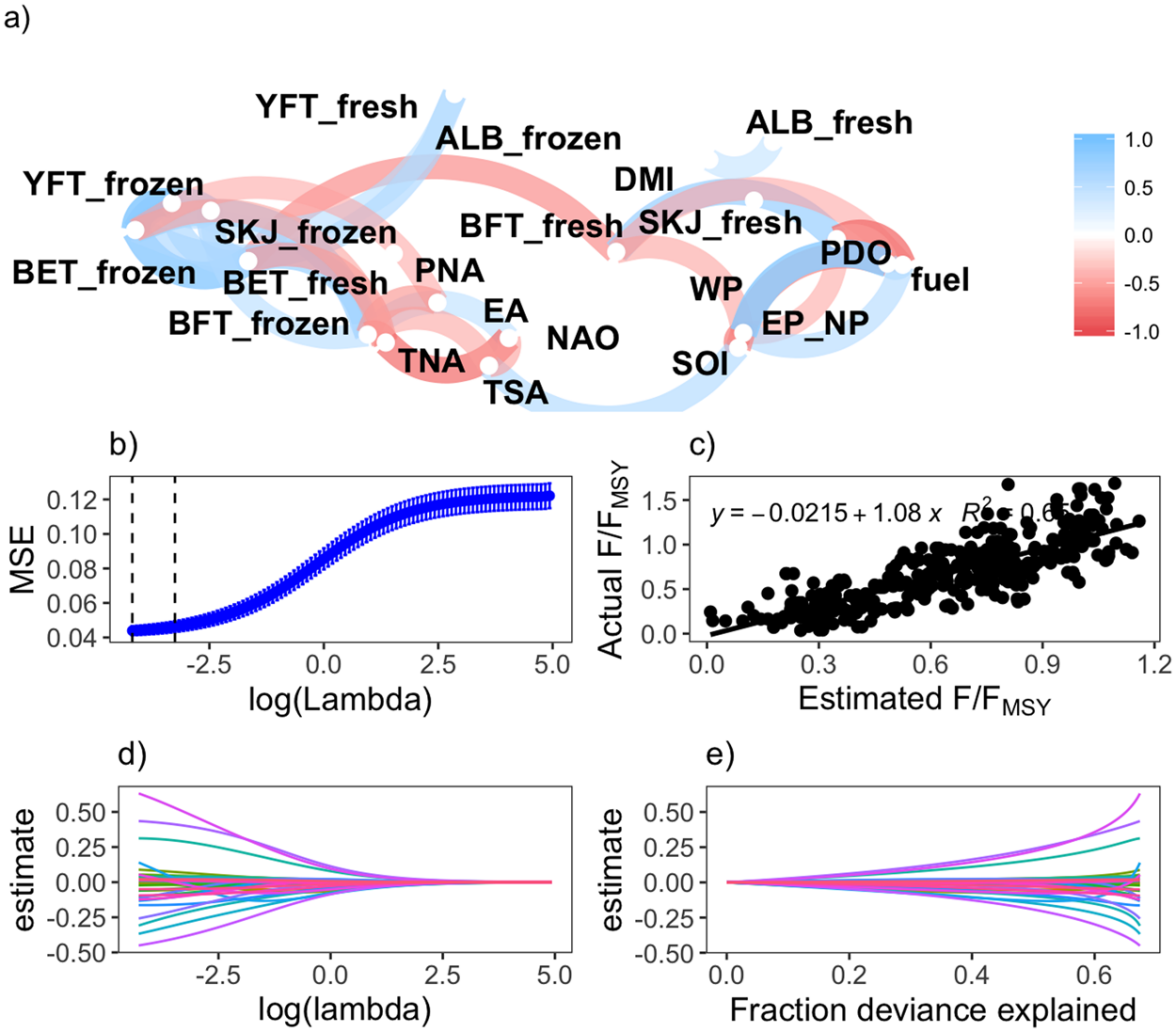
Model skill and cross-validation from the ridge regression analysis. (**a**) Pearson’s Correlation between feature variables, the plot uses clustering and the closer the variables are to each other the higher the relationship. While the opposite is true for widely spaced variables. The colour and thickness of the line represents the direction of the relationship and the strength. (**b**) Scatterplot to fit actual and estimated F/F_MSY_ for global tunas. (**c**) Mean – Squared Error (MSE) versus log ($\lambda )$ to show the cross-validation (CV) curve (blue line) with upper and lower standard deviations along the $\lambda$ sequence. The left most vertical line occurs at the CV minimum and the right vertical line is the largest value of lambda such that the error falls within one standard error of the minimum. (**d**) Estimates of the coefficients versus $\lambda$. (**e**) Estimates of the coefficients versus deviance explained (Overall deviance explained 68%).

To investigate the vulnerability of tuna stocks to short-term drivers of change we examined the effects of key economic, social and environmental variables using ridge regression to account for multi-collinearity of these variables. Correlations of note were found among variables, which were both positive and negative based on Pearson’s correlation (Fig. 2a). The majority of the tuna species prices were positively correlated (blue line) with some groups clustered together (bluefin and yellowfin tuna), while yellowfin, skipjack and albacore, for the most part were in individual clusters. On the other hand fuel price was not significantly correlated with any of the prices besides a significant negative correlation between fuel price and fresh skipjack price (SKJ_fresh), fresh bluefin (BFT_fresh).

The lack of correlation between fuel price and fish prices is perhaps due to de-trending of the inflation bias effect before conducting the analysis, as^30^ shows that both fuel and fish prices indices depict upward trends. The negative relationships between the fresh fish prices and fuel price is interesting because falling fresh fish prices (generally from near inshore coastal domestic fleets) could be related to the balance of demand, production and competition (see^31^). In terms of environmental indices, the El Niño Southern Oscillation Index (SOI) was negatively correlated with East North Pacific index (EP_NP) and Pacific Decadal Oscillation index (PDO), and clustered with a positive correlation with the Tropical South Atlantic index (TSA). In contrast NAO was negatively correlated with both TSA and Tropical North Atlantic index (TNA).

To estimate the role of these multicollinearity indices on F/F_MSY_ a ridge regression model was developed. The results from the ridge regression were plotted as a trace plot (described in Materials and Methods) where the predictor coefficients are plotted against $\lambda$ to simultaneously explain the greatest fraction of deviance and reduce the effects of collinearity (Fig. 2d,e). Caution is required if future conditions have not been seen in the historic observations (which is the case here) as this will limit the predictive skill of any model. As an example consider fuel prices, which have continued to increase at the same time as current management is aiming to recover stocks to levels that will support MSY (a level which has not been seen in the past). Therefore future costs and catch rates will be different than those used to fit the regression hence why de-trending the economic variables was of the utmost importance. Results from the 10-fold cross-validation produced a minimum $\lambda$ of 0.015 and a MSE of 0.04 was obtained (Fig. 2b) and used to test the model (coefficients are presented in Table 1) on the test data set, these estimates were plotted against actual values and gave an *R*^2^ of 0.65 (Fig. 2c) demonstrating its robustness for the analysis of potential trade-offs given uncertainty.

**Table 1:** Estimates from the Ridge regression analysis.

Variable	Coefficient
(Intercept)	0.766
fuel price	0.015
frozen price	−0.121
fresh price	−0.038
shannon wiener indices	0.017
human development index	0.074
Stock East Atlantic	0.149
Stock East Pacific	−0.105
Stock Indian	−0.140
Stock NorthAtlantic	−0.135
Stock NorthPacific	−0.261
Stock SouthAtlantic	0.400
Stock SouthPacific	−0.505
Stock SouthernOcean	0.487
Stock WestAtlantic	0.033
Stock WestPacific	−0.104
Species bigeye (BET)	0.273
Species bluefin (BFT)	−0.294
Species skipjack (SKJ)	−0.394
Species yellowfin (YFT)	−0.031
Gear gillnet	−0.013
Gear longline	0.006
Gear purseseine	−0.008
Gear trap	−0.066
Gear troll	−0.028
East Atlantic index (EA)	−0.041
Western Pacific index (WP)	−0.005
East North Pacific index (EP_NP)	0.055
Pacific North American index (PNA)	0.027
Dipole Mode index (DMI)	0.041
Tropical North Atlantic index (TNA)	−0.112
Tropical South Atlantic index (TSA)	−0.033
Pacific Decadal Oscillation index (PDO)	−0.025
Southern Oscillation Index (SOI)	0.054
North Atlantic Oscillation index (NAO)	0.042

### Differences in Sensitivity

**Figure 3:**
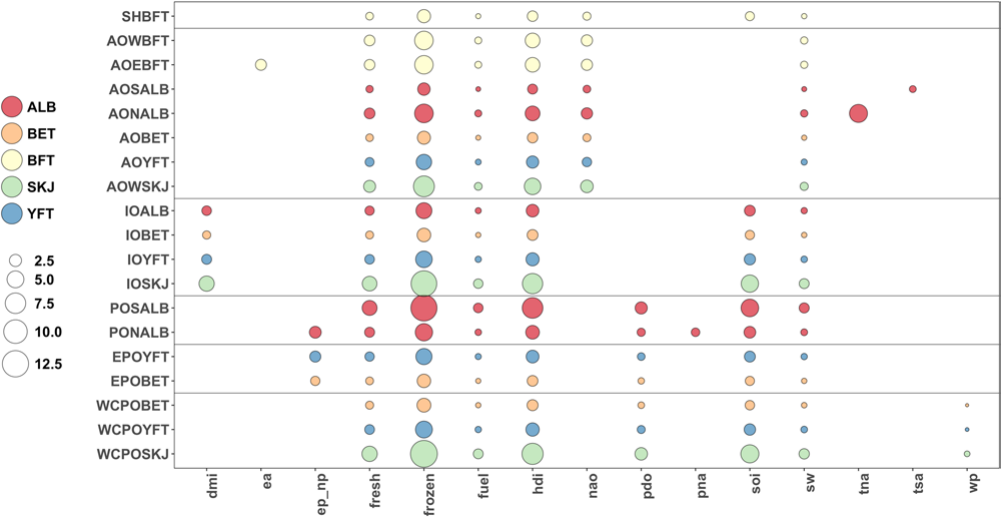
Elasticities by stock from the result of a 25% increase decrease in any of the 15 feature variables. The size of the bubble represents the resultant % change in F/F_MSY._ The colours represent the tuna species and the horizontal lines the tRFMO groupings (see Table 2 for the descriotion of the acronym).

Each sphere in Fig. 3 represents a different predictor effect on F_MSY_ for a particular tuna stock, a 25% increase or decrease, and the magnitude (size of the sphere) can be interpreted as the % change in F/F_MSY_. For example, when observing South Pacific albacore, a 25% increase in frozen price results in a 12% change in F/F_MSY_. Sensitivity to frozen price dominates, and appears to prominently affect the western central Pacific skipjack fleets with over a 13% change in F/F_MSY_. A 25% increase in HDI showed a notable change in F/F_MSY_ especially for the poorer regions of the world, up to 8%. This coincides with the findings in Fig. 1 and the “Trends in F/F_MSY_ and HDI” section (see above). As an approximate indication of the resulting changes of F/F_MSY_ on potential changes in % yields, our findings (Figure S2) depict that a 10% increase/decrease in F/F_MSY_ results in between a 3.7 and 8.8% increase/decrease in yield across all stocks. However, much larger reductions in F/F_MSY_ could lead to greater reductions in yield in the long term.

Fuel price was also dominant predictor of F/F_MSY_ across all stocks, with a 25% change in fuel price resulting in a 1.6% max change in F/F_MSY_, providing a positive link between the money spent and invested by a fleet, and the quantity of fish landed. Although this is a small increase and probably the offset effect of favourable frozen tuna prices and increases in technical efficiency, this can, however, have positive or negative effects on the stocks, i.e. such an increase in fuel price could have a large effect on the stock by reducing fishing mortality but quite the opposite effect from a drop in fuel price if not properly regulated. Either way, this substantial effect could be detrimental to the industry and the resource, or both. Many small-scale operators (e.g. the pole and line fleets) perhaps would have less opportunities for social change i.e. potentially a decline in fleet size or diversity (in terms of fishing areas and/or species) that could in turn have lasting effects in terms of food security for some coastal communities^32^. Longlining for tuna is on average up-to four times more fuel intensive per ton of catch than purse seining^33^ but the difference is very much smaller than that in specific fuel consumption per ton of catch, because of increases in fish prices for the better quality product. With much of the industry worldwide supported by government’s subsidies for fuel (in the western central Pacific alone worth in excess of US$335 million) (see ^34^), a price drop in fuel costs could lead to harmful and wasteful fishing practices. Therefore controlling fishing effort levels in the future via competitive fuel pricing and/or controlled market incentives such as encouraging the use of fuel-efficient technologies will be of great importance to global tuna fleets. In contrast, the species price effect resulted in a negative coefficient (both fresh (4%) and frozen (13%)), which is counter-intuitive to the expected behavior of fishers. Production sensitivities are usually positive; a higher price (*Ceterus paribus*) will lead to increased production. Although an elastic price effect of demand may occur whereby a moderate increase in catch will result in a substantial decrease in price^35^. However, in the case of purse seine fishers, it may be that the fishers target a higher abundance of fish even if the price is lower, therefore with overall higher total benefit. Fresh and frozen prices were included in this model to capture the dynamics of the sashimi and cannery markets, but maybe at the time of fisher decision-making the difference in price between fresh and frozen may not be relevant and therefore a composite measure of price would have been more appropriate proxy. Further, the quantity of frozen tuna can potentially be controlled in deep freeze and the quantity adjusted to market demand. It is also important to note that the fishing mortality on most tunas has increased^19^, which could also explain the negative effect.

The climatic effect, El Niño (SOI) had a large effect on F/F_MSY_, a 25% increase in the index resulted in a 5–6% increase in F/F_MSY_ for skipjack in the Indian Ocean and western Pacific Ocean which approximately equates to 53000t (8.3% yield change) and ~145000t (8.5% yield change) increase respectively. Previous studies have shown that tuna catches increase during the warmer periods of El Niño in the western Pacific^36^ and Indian Ocean^37^. Greater tuna biomass in these years is thought to supported by warmer water masses, which are their preferred habitats, perhaps also contributing their higher catch rates. For example^38^, showed that the western central Pacific purse seine fleet based their short-term decision-making by tracking sea surface temperature^39^. The Indian Ocean dipole index (DMI) also showed positive effects; with a positive index there was a significant increase in exploitation status for skipjack (4%) and yellowfin (1.5%), it is thought that some positive events coincide with El Niño events^40^. The NAO index, however, appears to show that it affects the exploitation of eastern and western Atlantic bluefin tuna, Northern Atlantic Albacore and western Atlantic skipjack (a 2–3% change in F/F_MSY_ from a 25% increase in NAO index). A positive anomaly in the NAO leads to a higher exploitation ratio. A positive NAO is the result of an Azores high and an Icelandic low, which results in an increased pressure gradient that causes westerlies to intensify and cold air to drain off the North American continent. Bluefin prefer cooler sea surface temperatures and frontal zones that give rise to food availability and survival for larvae^41^.

We used the Shannon Wiener index variables to investigate the diversity of the tuna fleet’s operations and as a proxy for communication and fishing efficiency, and this showed significant effects on F/F_MSY_. A positive coefficient reflects an increase in the spatial extent of operations and would be expected to result in an increase in mortality on the stock. Several authors have documented the dramatic changes in technology associated with the global tuna fishing fleets^42–44^, and how the use of new technologies by fishers (i.e. communication with other fishers) promotes information sharing leading to the most productive fishing grounds^45^ especially as stocks decline in size. Sophisticated FADs used during purse seine fishing determine the position and biomass of tunas below. Therefore the fishing operations may appear more spread out hence the positive coefficient. A 25% change in Shannon Weiner diversity indices resulted in a 2% increase in F/F_MSY_ for the western central Pacific fleet fishing on skipjack tuna.

## Conclusion

Tuna represent an iconic aquatic species that are important to many nations worldwide, not only for employment or economic returns from fishing, but are socially and culturally integral to local coastal communities as well as for the ecosystem. Our analysis has demonstrated how correlated social, economic and environmental variables can be combined in a simple model that can help to assess vulnerability to overexloitation and thus allow time for preventable management action.

Fisheries management has progressed over the course of the 20th century, but given the large proportion of stocks that are depleted or over-exploited^7^, the threat to many coastal communities, and the increasing number of marine species that have been lost or listed as endangered^46^, there is still a clear need for improved management. Our approach is necessarily simplified in that we analysed trends relative to fixed references points from stock assessment outputs. In reality changes in stock structure and environment will change F_MSY_ (and also MSY and B_MSY_). Future work could aim to address these influences in more depth by integrating environmental variables into dynamic population models.

## Materials and Methods

### Data

There were several key sources of data used to build the model: stock assessment outputs, spatialy explicit catch and effort data and associated indicators of fisheries spatial spread, fish landed value and fuel price data, and environmental indicators previously been found to be important for tuna stock variability (the Southern Oscillation Index (SOI) in western central Pacific^35^ and the North Atlantic Oscillation Index (NAO)^47^). Assessing exploitation status relative to sustainable reference points is a key aim of stock assessments. F/F_MSY_ time series data were acquired from the RAM legacy stock assessment database^48^ by year, stock (see Table 2) and species (skipjack (SKJ), yellowfin (YFT), albacore (ALB), bluefin (BFT) and bigeye tuna (BET)) (http://ramlegacy.org).

**Table 2:** A summary of all available F/FMSY data on tuna stocks by management organisation (tRFMO) (indicated by a tick).

Stock	tRFMO	Data
***Stocks in the Eastern Pacific Ocean***		
*EPOBET Bigeye Tuna*	IATTC	✓
*EPOYFT Yellowfin Tuna*	IATTC	✓
*EPOSKJ Skipjack Tuna*	IATTC	✖
***Stocks in the Western and Central Pacific Ocean***		
*WCPOBET Bigeye Tuna*	WCPFC	✓
*WCPOYFT Yellowfin Tuna*	WCPFC	✓
*WCPOSKJ Skipjack Tuna*	WCPFC	✓
***Pacific-wide Stocks***		
*PONALB North Pacific Albacore*	ISC	✓
*POSALB South Pacific Albacore*	WCPFC	✓
*POBFT Pacific Bluefin Tuna*	ISC	✖
***Stocks in the Atlantic Ocean***		
*AOBET Bigeye Tuna*	ICCAT	✓
*AOYFT Yellowfin Tuna*	ICCAT	✓
*AOESKJ Eastern Skipjack Tuna*	ICCAT	✖
*AOWSKJ Western Skipjack Tuna*	ICCAT	✓
*AONALB Northern Albacore Tuna*	ICCAT	✓
*AOSALB Southern Albacore Tuna*	ICCAT	✓
*AOMALB Mediterranean Albacore Tuna*	ICCAT	✖
*AOEBFT Eastern Atlantic and Mediterranean Bluefin Tuna*	ICCAT	✓
*AOWBFT Western Atlantic Bluefin Tuna*	ICCAT	✓
***Stocks in the Indian Ocean***		
*IOBET Bigeye Tuna*	IOTC	✓
*IOYFT Yellowfin Tuna*	IOTC	✓
*IOSKJ Skipjack Tuna*	IOTC	✓
*IOALB Albacore Tuna*	IOTC	✓
***Southern Hemisphere Stocks***		
*SHBFT Southern Bluefin Tuna*	CCSBT	✓

### Covariates of exploitation status

The global tuna catch and effort databases from tuna RFMOs (which contains aggregated commercial logbook data of catch and effort by fishing gear) were used to develop a time-series of commercial purse seine, baitboat, longline, gillnet, troll and trap (area/stock specific – see Table 3) effort estimates for fleets operating between 1990 and 2014. The data collected for each country included an effort variable and in most cases for purse seine/baitboat/trap/gillnet was represented by the approximated fishing duration as number of trips/days/hours fished (or at sea) per one-degree cell. For longlines/trol this was a mixture of number of sets, hours and hooks per five-degree cell (longlines), year. The longline and purse seine fishing activity was converted to number of days at a scale of 1° (purse seine) or 5° (longline) cell by applying different conversions factors to the different effort types in order to produce a common variable, days fished (although days fished for purse seine and number hooks for longline could have been used, however, these data were available and formatted as part of on-going research in tandem and thus was considered practical) (see Table 3).

**Table 3:** Tuna RFMO online data conversion to days fished.

tRFMO	Fleet	Conversion to days	Scale	Reference
WCPFC	**Purse seine**	Already days	5 × 5	
	**Longline**	Hooks and soak time to days	5 × 5	^60^
	**Baitboat**	Already days	5 × 5	
IOTC	**Longline:**			
	*ELL Longline (targeting swordfish)*	Hooks per set 1600 and soak time to days	5 × 5	IOTC website (number of hooks)^60^
	*FLL Longline Fresh*	Hooks per set 1200 and soak time to days	5 × 5	IOTC website (number of hooks)^60^
	*LL Longline*	Hooks per set 2750 and soak time to days	5 × 5	IOTC website (number of hooks)^60^
	*LLEX Longline (exploratory fishing)*	Hooks per set 2750 and soak time to days	5 × 5	IOTC website (number of hooks)^60^
	*SLL Longline (shark species)*	Hooks per set 1600 and soak time to days	5 × 5	IOTC website (number of hooks)^60^
	**Purse seine:**			
	*PS Purse seine*	Fishing and search hours converted to days	1 × 1	
	*PS Purse seine*	Sets converted to days an average of 0.8 (to include FAD)	1 × 1	(free school^61^ 0.5–0.65)^62^; (FAD 0.7–0.92)
	*PSS Small purse seine*	Trips to days 1 trip = 7–15 days - used 11	1 × 1	^63^
	**Baitboat:**			
		Trips to days 1 trip = 16 days	1 × 1	^64^
	**Gillnet:**			
		30–45 days per trip - used 30 days	1 × 1	^65^
ICCAT	**Longline**	Number of hooks IOTC conversions used for comparable gears	5 × 5	
	**Purse seine**	Hours converted to days	1 × 1	
	**Baitboat**	Already days		
	**Trap**	Trap day/days fished	1 × 1	
	**Trol**	Already days	1 × 1	
IAATC	**Longline**	Hooks per set 1865 with 19 hours soak time	5 × 5	^60^
	**Purse seine**	Sets per day average of 0.8	1 × 1	(free school^61^ 0.5–0.65)^42^; (FAD 0.7–0.92)
	**Baitboat**	Approximately 5 schools per day		^66^
CCSBT	Longline	Taken IOTC hooks per set 2750 and soak time of 22hrs	5 × 5	^60^
	Purse seine	Hours converted to days	1 × 1	
	Baitboat	Hours converted to days	1 × 1	

Spatial expansion of fisheries are known to occur as fisheries develop, as well as contracting at low population densities thus could provide a useful proxy for exploitation status. Shannon Wiener indices of fleet spatial behaviour by gear were used to estimate the spatial spread of fishing effort as has been previously used for tuna fleets^44^. This index is derived from information theory (knowledge transfer and represents the social variable) and measures the amount of order/disorder within a system; it is widely used in ecological research to study species diversity^49^. An index of zero indicates that only one area (1° or 5° cell) was visited within a specific fishing year. Unlike the study mentioned above, these data sets did not contain any information on vessel characteristics, trip ID or fleet composition in terms of numbers of vessels. Therefore the effort data were aggregated by year (*y*), gear (*g*) (longline or purse seine), organisation (*o*) (tRFMO) and stock (*s*) (i.e. Indian Ocean, Atlantic east or west etc…). An increase in the index describes how equally fishing effort is distributed across areas (*a*):

1 $${y}{{e}}_{{s}{{w}}_{{o},{s},{y},{g},{a}}}=-\,\sum _{{a}=1}^{A}-(\frac{{{E}}_{{o},{s},{y},{g},{a}}}{{{E}}_{{o},{s},{y},{g},{A}}})\mathrm{log}(\frac{{{E}}_{{o},{s},{y},{g},{a}}}{{{E}}_{{o},{s},{y},{g},{A}}}).$$

Economic theory suggests that fishers make their strategic choices based on changing stock biomass levels, management regulations (effort controls), market prices, and fuel costs. Ideally individual vessel cost data would be necessary to conduct a full bio-economic model; however much of these data are not available. As a result, several variables were used as surrogates, e.g. value as a proxy for economic viability and fuel price as a proxy for cost. US gulf prices (US$) per barrel were obtained from the US energy information administration (http://www.eia.gov/petroleum/data.cfm) averaged and adjusted for inflation using the IMF (average of three spot prices) index (https://knoema.com/IMFWEO2015Oct/imf-world-economic-outlook-weo-october-2015?tsId=1072230) relative a base year of 2014 and is known as the real price. Fisher skills, knowledge, and experience are expected to relate to the annual revenues of the target species of the fleet. Fresh and frozen prices were included to account for changes in the markets and hence targeting, i.e. when there is a shortage of fresh/frozen tuna the price increases. Price per metric kg data (Yen ¥) of the main target species, skipjack, yellowfin, albacore, bluefin and bigeye tuna fresh and frozen was acquired from Tsujiki market ex vessel prices from^50^ and the NOAA fisheries statistics website http://www.st.nmfs.noaa.gov/commercial-fisheries/market-news/related-links/market-news-archives/index). Average fish prices were calculated by year, which were subsequently converted to $US by applying historical conversion rates (https://www.oanda.com/currency/average) and adjusted (standardized) by the inflation factor for FAO fish price index (FPI) (http://www.fao.org/in-action/globefish/fishery-information/resource-detail/en/c/338601/) relative to a base year of 2014 to reduce bias in the data and is known as the real price.

The SOI and the NAO were obtained from the Australian government Bureau of Meteorology (http://www.bom.gov.au/climate/current/soihtm1.shtml) and (http://www.cpc.ncep.noaa.gov/products/precip/CWlink/pna/nao.shtml) respectively, to track the climatic effects. The SOI has been shown to be important globally (see ^51,52^) and has been thought to specifically affect tuna migrations and hence catchability particularly in the Pacific and the Indian oceans^37^ while the NAO has influenced Atlantic tunas^47^. Other area specific climatic indices were collected and combined to assess their effects on the 19 tuna stocks; these include the Indian Ocean Dipole Mode Indices (DMI), (http://www.jamstec.go.jp/frcgc/research/d1/iod/HTML/Dipole Mode Index.html), the tele-connection indices: East Atlantic (EA), West Pacific (WP), East Pacific-North Pacific (EP-NP), Pacific North America (PNA), and the Tropical North and South Atlantic Index (TNA and TSA) which were found at (https://www.esrl.noaa.gov/psd/data/climateindices/list/).

**Figure 4:**
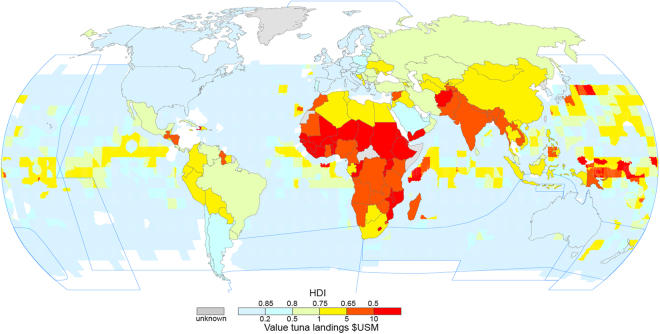
Comparison mapped nominal value of tuna harvest ($US M) in 2014^57,58^ compared with national higher development index (HDI) status for 2015^59^. Regional fisheries management organisations boundaries relevant to tuna fisheries are show by blue lines (see Supplement Figure S1).

To track the link with human development and stock status HDI is included in the dataset (http://hdr.undp.org/en/data). The data described above are presented in Figs 1 and 4. All data above were averaged by year and merged with the spatial indices to create the database used in the analysis.

### The model

When analysing such large and complex datasets correlation between dependent variables may mean parameter estimates are unstable and that the results do not reflect the actual relationship between the variables being studied^53^. Ridge regression is a modelling application that can deal with such issues of multi-collinearity and is an appropriate tool to model the economic, climatic and spatial diversity effects of the tuna fleets on their relative fishing exploitation status (F/F_MSY_). Since it was proposed^54^ ridge regression is one of the most widely used methods to tackle the issues of multi-collinearity. They suggested that there was potential instability in the OLS since OLS is heavily reliant on (*X′X*)^−1^. *X* is a square matrix of order (n × p) of centred observations ‘n’ on ‘p’ predictor (features), and *Y* is a n × 1 vector of centred observations on a response variable and *X’Y* is the correlation between *X* and *Y:*

2 $$\hat{{\beta }}={({X}{^{\prime} }X)}^{-1}({X}{^{\prime} }Y)$$

thus any change in *X* may lead to large changes in (*X*′*X*)^−1^ which may offer a better fit of the data (describing how well the model fits the observations) but it may have poor predictive power. This is because high multi-collinearity may lead to a high mean square error MSE in *β*, which implies $\hat{{\beta }}$ is an unreliable estimate of *β*. The authors suggested adding a term *λ* a constant value in order to stabilise the diagonal entities of (*X′X*)^−1^ and therefore the estimator is described formally as (*I*_*P*_ represents matrices of eigenvalues and eigenvectors = diagonal vector of ${\lambda }_{1\ldots \ldots \ldots \ldots }{\lambda }_{\rho }$):

3 $${\hat{{\beta }}}_{\mathrm{ridge}}={({X}{^{\prime} }X+{\lambda }{{{\rm I}}}_{{P}})}^{-1}({X}{^{\prime} }Y)$$

which places penalties on the $\beta ^{\prime} s$ which minimalizes the penalised sum of squares.

4 $$\sum _{{i}=1}^{{n}}{({{y}}_{{i}}-\sum _{{j}=1}^{{p}}{{x}}_{{ij}}{{\beta }}_{{j}})}^{2}+{\lambda }\sum _{{j}=1}^{{p}}{{\beta }}_{{j}}^{2}$$

where ${{\beta }}_{{j}}$ are the parameters constrained in the linear model by the constant ${\lambda }$. Therefore if ${\beta }$’s take on larger values they are subsequently penalised in the optimisation routine. It was suggested^55^ that ${\lambda }$ should be small enough so that the MSE of the ridge estimator is less than OLS estimator.

Choosing $\lambda$ has often been a difficult obstacle faced by an analyst as^54^ stated and subsequently invented a graphic called ridge trace to assist. Simply ridge trace is a plot of the ridge coefficients for a given level of $\lambda$ and the analyst selects the level of $\lambda$ at the point where the coefficients have stabilised i.e. introduces the smallest bias. However instead of arbitrarily choosing a value of $\lambda$ it would be more beneficial to select the tuning parameter on the basis of cross validation. The basic theory behind cross-validation is to split the data by removing a portion to build a model (the training set), then using the remainder of the data (the test set) to test the performance of the training set model by computing the mean square error and the minimum associated $\lambda$. The procedure is repeated k times (10 fold) by randomly partitioning different portions of the data in turn and predicting the test set k − 1. Each model is then assessed on the different subsets of the data it predicts and an average proportion predicted is compared with the observed data from each test set.

The final procedure is to refit the ridge regression model with the minimum $\lambda$ resulting from the cross validation on the full data set to obtain the coefficient estimates.

### The analysis

The ridge regression conducted for this study included 6 core continuous feature variables (i.e. fuel cost (“realfuel”), climate (“SOI” and “NAO”), spatial effects (“SW”), fresh and frozen price of the core tuna species (“realfresp” and “realfrozp”)). Categorical variables (i.e. stock, species, and gear (stock/region specific – i.e. No ‘TRAP’ gears for any other region apart from East Atlantic bluefin)) are encoded as dummy variables 1 or 0. There were 1107 rows and 35 columns in the dataset including the response variable. These data were split into a separate datasets of regressor and response matrices and each randomly further split into training (2/3) and test sets (1/3). A ridge regression model was fit to the training set in order to determine a value of the tuning parameter $\lambda$.

**Figure 5:**
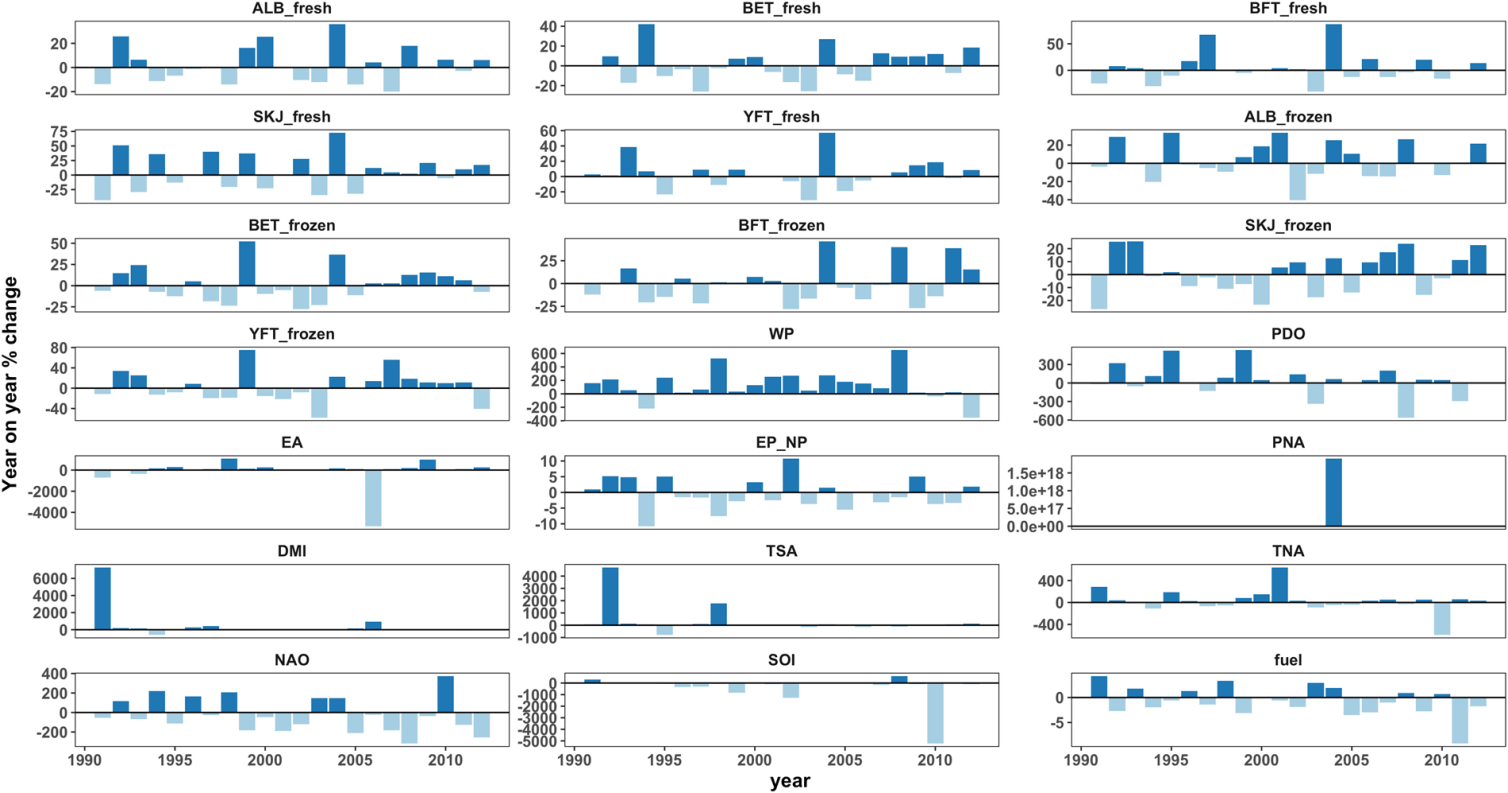
Year-on-year % changes for the main feature variables.

The marginal effects were calculated on the continuous predictor variables in order to understand their effects on F/F_MSY_ i.e. a 25% change in *X* gives a % change in *Y*. while all other variables were held constant. Twenty five per cent seemed to represent a reasonable number given the year on year changes on the variables as presented in Fig. 5.

The glmnet packages were used to perform the ridge regression analysis in R^56^.

### F/F_MSY_ v HDI

HDI were obtained from the United Nations Development Programme for the years 1990–2012 (http://hdr.undp.org/en/data) for all available countries. Here we compared countries average HDI score within a stock region (Table 2) with F/F_MSY_ status for that particular stock within two time periods (1990–2001 and 2002–2012) using a simple linear regression. Figure 4 for example displays the average HDI changes by country for 2012.

### Data availability

The data supporting the findings of this study are available from the corresponding websites links embedded in this section.

## Electronic supplementary material

Supplementary Information
