# Supplementary material for: Predicting global tuna vulnerabilities with spatial, economic, biological and climatic considerations
Source: Sci Rep. 2018 Jul 12;8:10572. doi: 10.1038/s41598-018-28805-z (PMC6043545; doi:10.1038/s41598-018-28805-z)
Supplement: Supplementary file 1 — Supplementary Information [file 41598_2018_28805_MOESM1_ESM.pdf]

Title: Predicting global tuna vulnerabilities with spatial, economic, biological and climatic considerations.

Author Affiliation:

Alex Tidd[1, 2, 3], Julia L. Blanchard[1, 2], Laurence Kell[4] and Reg A. Watson[1, 2]

[1]Centre for Marine Socioecology, University of Tasmania, GPO Box 252-49, Hobart, Tasmania 7001, Australia.

[2]Institute for Marine and Antarctic Studies, Centre for Fisheries and Aquaculture, University Tasmania, Private Bag 49, Hobart, 7001 Tasmania, Australia.

[3]Galway-Mayo Institute of Technology (GMIT), Old Dublin Road, Galway, Ireland.

[4] Sea++, The Hollies Hall Farm Lane, Henstead, Suffolk NR34 7JZ, UK.

Supplementary Information

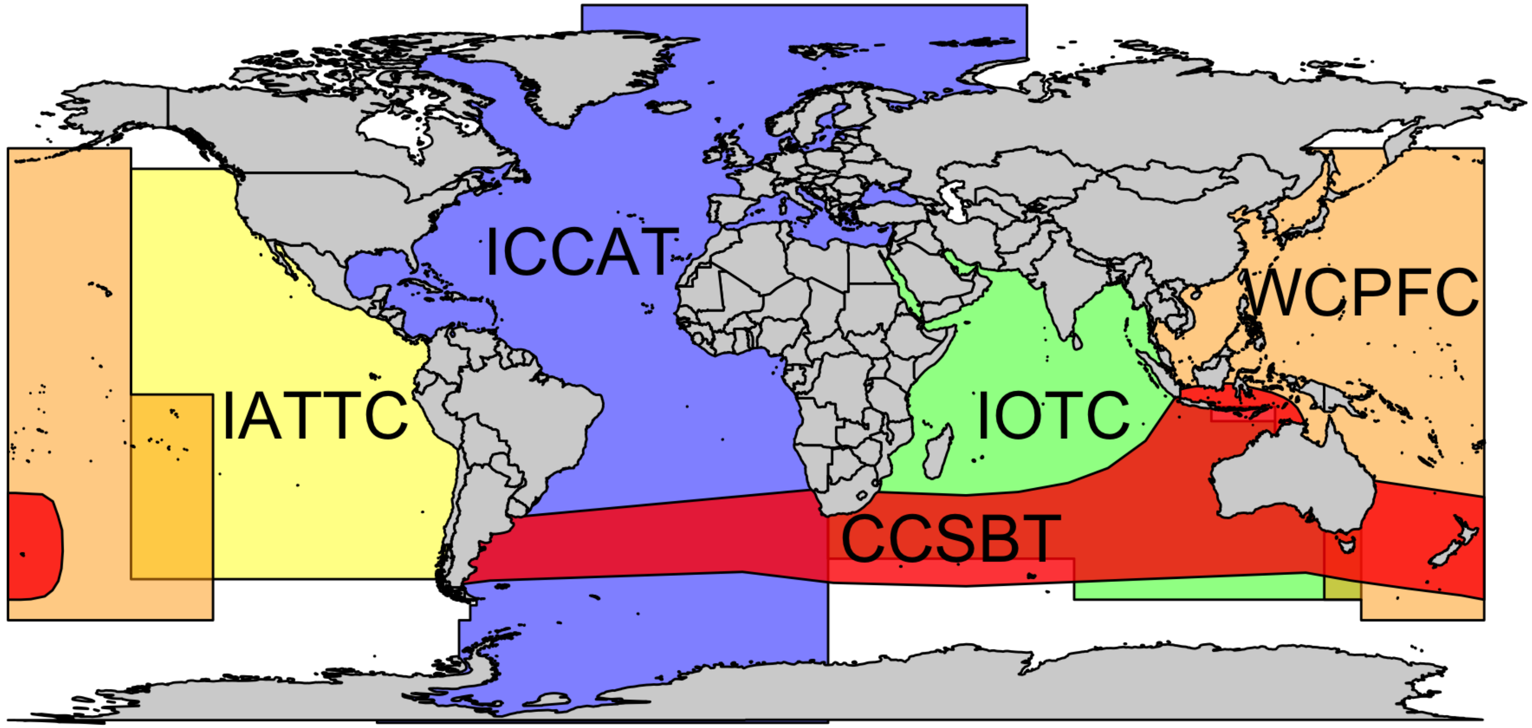

Figure S1 Map of tRFMOs (colours differentiate tuna Regional Fisheries Management Organisation regions). There are 5 organisations, (International Commission for the Conservation of Atlantic Tunas (ICCAT), Indian Ocean Tuna Commission (IOTC), Western and Central Pacific Fisheries Commission (WCPFC), Inter-American Tropical Tuna Commission (IATTC) and Commission for the Conservation of Southern Bluefin Tuna (CCSBT) plus (ISC), the International Scientific Committee for tuna) that assess and manage 23 of the highly migratory stocks of the 7 globally important oceanic tuna species). The tuna industry within each (RFMO) is often split into 2 main sub industries,

longlines and purse seines. The purse seine fishery which mainly targets skipjack (*Katsuwonus pelamis*), and yellowfin tuna (*Thunnus albacares*) between 20°N and 20°S, while bigeye tuna (T. obesus) are also captured as bycatch by purse seiners fishing on fish aggregating devices (FADs) for the low valued canned tuna industry. Conversely, the longline industry specifically targets bigeye tuna, yellowfin tuna, albacore tuna (*Thunnus alalunga*) and bluefin tuna (Atlantic (ICCAT): *Thunnus thynnus*; Southern (CCSBT): *Thunnus maccoyii*; Pacific (IATTC; WCPO) *Thunnus orientalis*, mainly for the highly priced sashimi market.

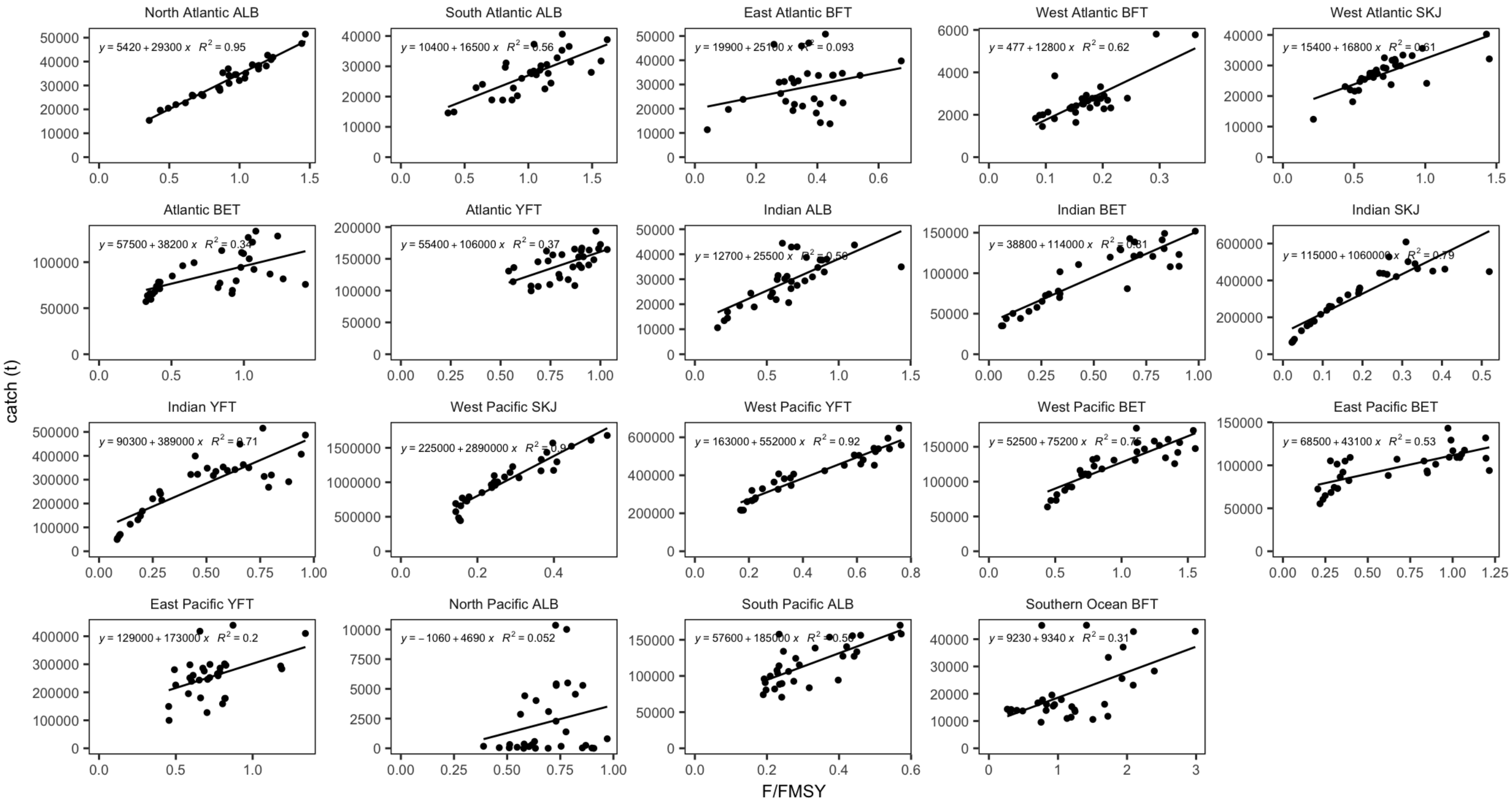

Figure S2 Translating from changes in F/Fmsy to changes in yields. Ideally the input data to carry out global tuna stock assessments would be used to run short and medium term projections to assess risk and effects of changes in F/FMSY on landings along with their associated

uncertainties and nonlinear responses. However, since we have assessment outputs only, a rough approximation of the relationships between historical yields and F/FMSY provides a range of potential values across stocks.
